# A shout‐out to middle authors: Opportunities for achieving clarity in editing

**DOI:** 10.1002/bco2.90

**Published:** 2021-05-28

**Authors:** John W. Davis

**Affiliations:** ^1^ Editor, *BJUI Compass*

For the May 2021 issue of *BJUI Compass,* I want to give a shout‐out to middle authors of our manuscripts. Obviously, we need first authors to take the lead on collecting data and writing up a project and we need last/senior authors for guidance, design and funding. But we often need strong contributions from middle authors who recruit patients to studies, assist in design/execution of trials/studies, and provide critical editorial work for drafting manuscripts. I was inspired to write this editorial from two different experiences. On the academic front, my research team had a manuscript reviewed by a journal and offered major revisions. They were tough ones—requiring additional analyses and statistical work. One of our strategies for revisions was to invite another urologic oncology fellow to the team to give us a fresh look at the manuscript and help us with the revision steps and getting the paper not only revised but back to word limits. The revised version completed peer review and is now published.

**FIGURE 1:**
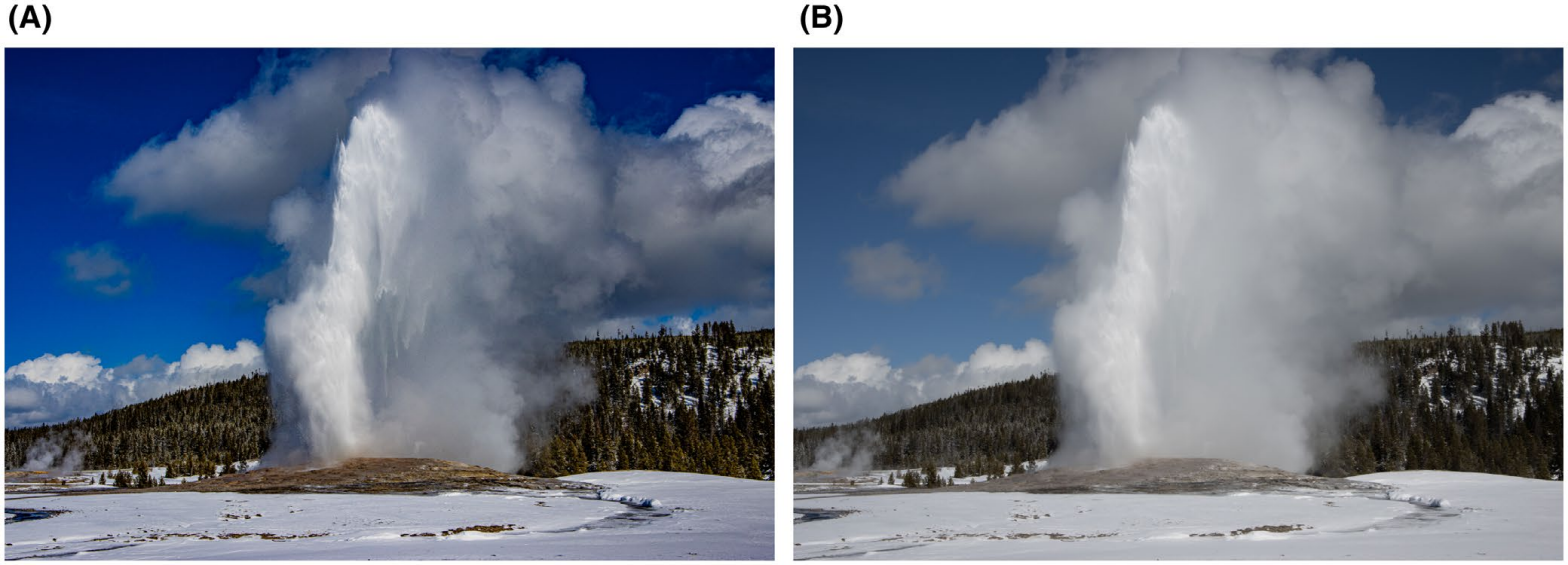
(A) The famous “Old Faithful geyser in Yellowstone National Park in Wyoming. The name derives from its highly predictable eruption timing—every 44‐120 minutes. This picture was edited in Adobe Lightroom to adjust: enhanced shadows, enhanced contrast, no highlights, reduced white noise, enhanced vibrance, boosted blue palate, and enhanced Dehaze—the latter to bring out the clarity in the water jets and droplets relative to the surrounding steam. Each eruption spits out 3700 to 8400 gallons of water. (B) By contrast to Figure 1A, this is the original, unedited version with more shadowing around the trees, a duller sky (but not bad), and more blurring of the water from the steam blowing out of the geyser. By analogy, we want our middle authors to help us edit our fine work and provide clarity in writing so our readers can enjoy the most detail and efficiency in their reading and learning

**FIGURE 2:**
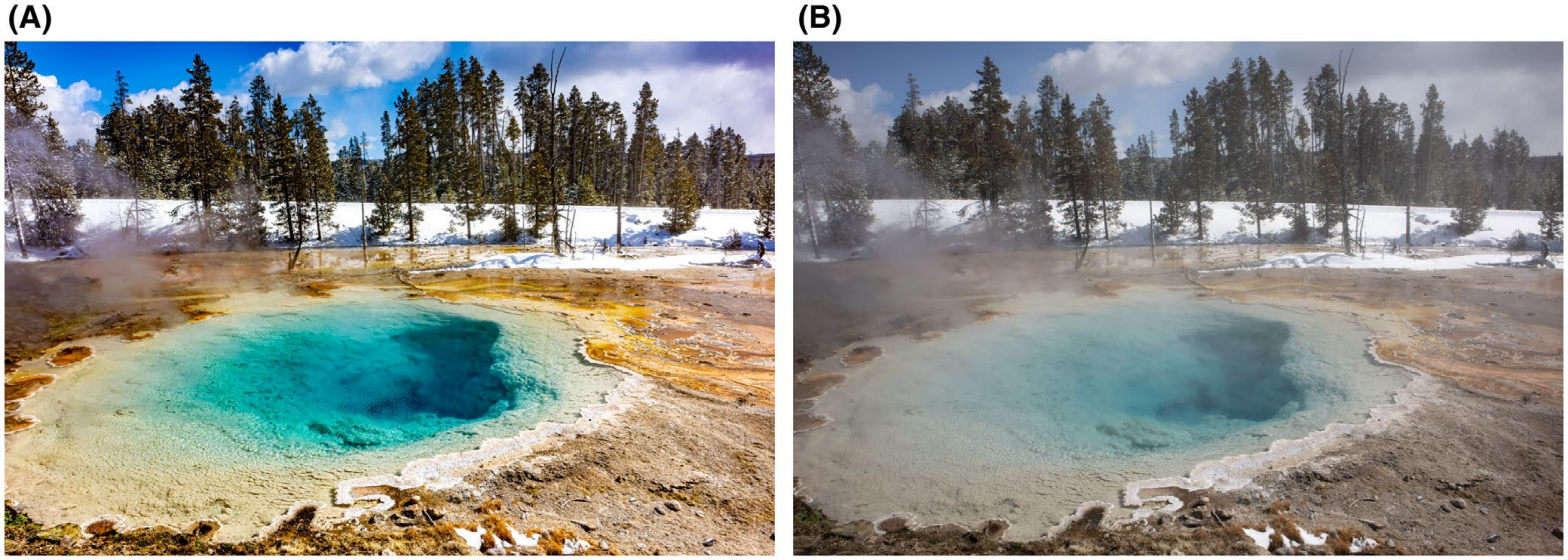
(A) A hydrothermal area of the Fountain Paint Pot Trail in Yellowstone National Park. This volcanic area features mudpots, geysers, hot sprints and fumaroles. This is Silex Spring—very hot water with clear blue water and a nearby thermophile mat to the right—an area of bacteria that can survive hot temperatures. (B) As with Figure 1A/B, you can see the pre/post editing with Adobe Lightroom—more steam and less colour definition from the water and the thermophile mats

Another point of inspiration came from a recent trip I made with my family—yes, a trip during pandemic times! Many of the photographs I’ve published with this editorial series were from pre‐pandemic academic trips—I have a large inventory, but in theory could run into slim pickings if this pandemic keeps dragging on. Our family loves to ski at Jackson Hole, Wyoming, and rather than sit out another season, we made the journey with a long drive from Houston to Wyoming—a solid full 2 days of driving each way (I’m not quite ready for aeroplanes). In addition to the challenging skiing at Teton Village, another opportunity for winter sports is snowmobiling in Yellowstone national park—famous for its geysers and multiple volcanic activities. The whole park is actually a giant volcanic caldera—last erupted over 630,000 years ago but in theory could blow again. In Figure 1, I will demonstrate a fun photography editorial trick from Adobe's Lightroom photo‐editing software. It is called the “dehaze” feature and it does an amazing job of getting rid of hazy appearing backgrounds—especially grey/dull skies, or city smog. Specific to geysers—it's a fun feature to tone down a lot of the steam coming out of these volcanic entities. Figure 2 shows another example from a hydrothermal pool—again with editing of the steam and colours for a more dramatic look. The analogy for our middle authors is that their editing efforts and fresh look at our papers can be effective methods for improving a manuscript. A primary writer can certainly edit and revise a paper, but sometimes another set of eyes can take it to the next level. These collaborative efforts can pay off in peer review. As Prokar Dasgupta, previous editor of the BJUI, used to say at lectures on publishing: ***“You have one good chance to make a good first impression.”***

**FIGURE 3:**
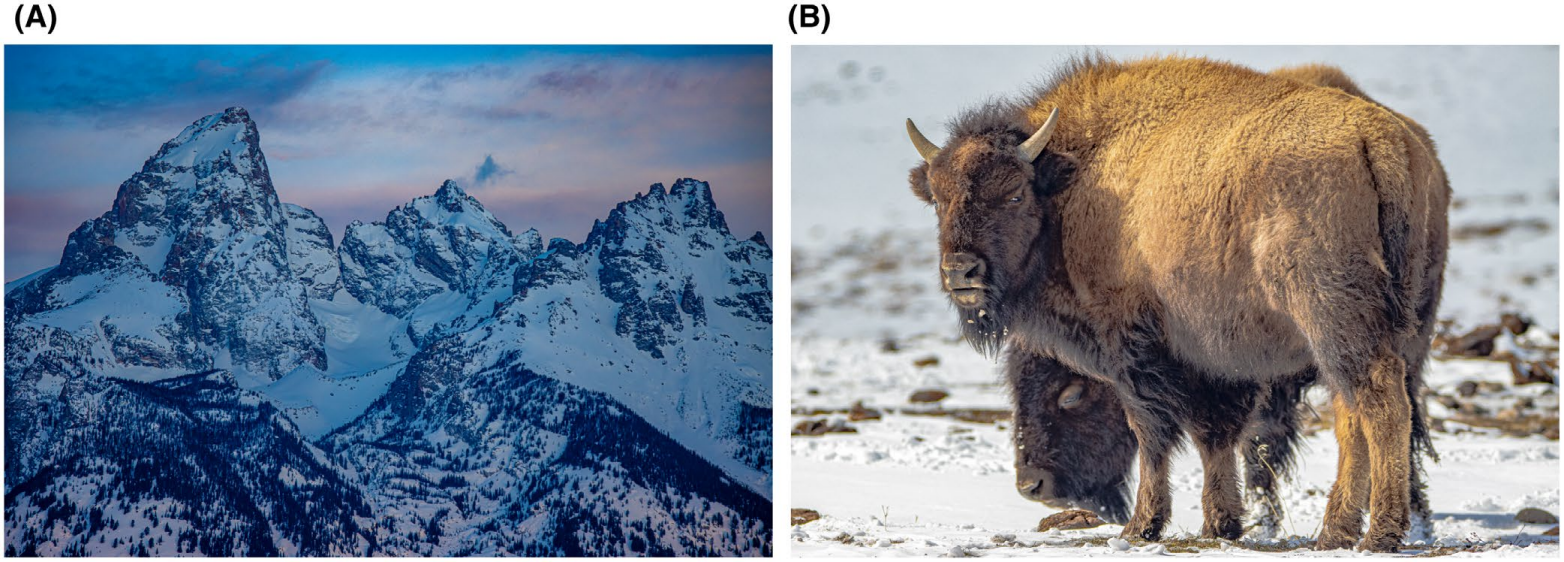
(A) The Grand Teton Range in Wyoming at sunrise. These mountains are surrounded to the East by a flat/valley area that used to be a river bed—thus allowing distant viewing. This picture was taken at 100mm focal length—an example of how telephoto lenses can be effective for landscapes as well as super wide images. (B) The American Bison herd in Yellowstone National Park—a fragile herd of around 4,800 that can brave the harsh and long winters of Northern Wyoming. We often see the herds near volcanic activity, as the heat keeps some of the snow off the grass. Springtime is a bit of a race for time as the herd must conserve energy and wait for the Spring thaw to have better access to grazing

Figure 3—more fun shots from the Tetons and Yellowstone—a must visit place. On to this month in the *BJUI Compass:*

- **To the Journals** … We have two nice reviews in this issue. The paper by Aluwini et al^1^ is a consensus effort around the diagnostics and initial treatments for stage M1a prostate cancer. This group was in favour of PSMA imaging as a standard but for treatments the group review several known strategies around androgen deprivation and lesion directed therapy but lacked consensus. Although this is not a traditional review, this type of paper effectively highlights the dilemmas in this space and where future research needs to focus. The review by Russel et al^2^ is a traditional systematic review in bladder cancer. They focused on socioeconomic status as a predictor of survival but could also show relationships with modifiable risk factors such as treatment delays. A hint for future authors—this authorship group has an open access agreement in place that covers article publication charges—be sure to check the Wiley website to see if your institution has one (see our web page and follow the links to Wiley Open Access).
- **To the Clinic** … We have three clinical utility papers for your interest—focused efforts on prostate cancer pathology correlates to outcomes,^3^ a practical study asking the question as to whether or not the prostate screening digital rectal exam matters any more when PSA and MRI are known,^4^ and a detailed comparison of MRI accuracy metrics judged by radical prostatectomy findings across different anatomic zones.^5^ This latter paper is useful in that anterior lesions had lower sensitivity for MRI, but the authors do question whether negative findings need additional corroboration (biopsy, other imaging, etc).
- **To the Drawing Board** … We have two papers in this section. Wei et al^6^ looked at a well characterised population of men with mpMRI and transperineal biopsies and compared multiple risk calculators. One outperformed two others. I won't steal their thunder—download the article to find the winner. Martos et al^7^ looked at PSA trends in patients treated with HoLEP and noted key nadir levels and follow‐up trends.
- **To the Future** … Rounding out our issue is an innovation themed paper from Silagy et al.^8^ This paper is a bit of a “looking back/history lesson” but is a nice illustration of surgical innovation. As you may recall, robot‐assisted radical prostatectomy took the lead in robotic urologic surgery in the early 2000s. There was a bit of a delay getting this approach expanded to the partial nephrectomy operation, and it took some ingenuity to address the main concern of solving warm ischemia time limitations. Of course, laparoscopic partial nephrectomy was described but required extraordinary surgical skills and highly selected cases. This paper details the history of the “sliding Weck Clip” technique that many of the early robotic pioneers figured out and used to streamline this operation with feasible warm ischemia times and enhanced ability to train surgeons on the technique and tackle more advanced tumours. As this article details, the sliding Weck clip was an Australian concept used in open and laparoscopic techniques and later mainstreamed into robotics. Figure 1 shows a beautiful artist's rendition of this now‐famous technique. Congratulations to the authors and thank you for your clever contribution.
